# Employment of Veratria in Acute Diseases of the Chest

**Published:** 1860-01

**Authors:** 


### *Employment of Veratria in Acute Diseases of the Chest.*

M. Aran has called the attention of practitioners to the remarkable effects produced by the internal use of veratria in febrile diseases, and especially pneumonia. In the Sardinian Medical Gazette an article has appeared, in which Dr. Ghiglia, without any knowledge of M. Aran's researches, recommends the use of veratria in the same circumstances, except that he never employs this alkaloid alone, but associates it almost always with opium, sometimes in the form of pill, sometimes as a syrup. The dose of veratria is five millegrammes (.077 of a Troy grain) in a pill, with the same quantity of opium, and the number of pills to be taken in the twenty-four hours varies from six to seven, and even twelve, according to the circumstances. In this dose, according to M. Ghiglia, vomiting rarely occurs, but nausea and the other depressing effects of veratria are present. The results obtained by M. Ghiglia in certain cases of pneumonia, bronchitis, and broncho-pneumonia have been sometimes most remarkable, and the following are the results arrived at by this author:—"1. The inflammation of the respiratory organs, when they have arrived at such a period as to produce disorganization of the parts, are not improved by the use of veratria. 2. The action of this substance is the more favorable in proportion as the disease is more recent. 3. The tolerance is very various, according to individual habits, and perhaps also according to certain peculiarities which are not well understood. 4. The more easily the tolerance ceases the more marked is the depression. 5. Veratria is, in many respects, a preferable medicine to others which are more constant in their action but less easy to take. And 6. It is perhaps prudent, in severe inflammations of the respiratory organs, to order a few bleedings before prescribing the veratria.—*Bulletin General de Therapeutique*, *January* 30, 1859.

